# P-1208. Multi-year Analysis of Respiratory Viral Dynamics at a Los Angeles County Academic Health System Reveals Significance of Rhinovirus in Young Children with Severe Respiratory Illness

**DOI:** 10.1093/ofid/ofae631.1390

**Published:** 2025-01-29

**Authors:** J R Caldera, Tawny Saleh, Trevon Fuller, Shangxin Yang, Karin Nielsen-Saines

**Affiliations:** David Geffen School of Medicine, University of California, Los Angeles, California, USA, Los Angeles, California; Division of Preventive Medicine, Department of Medicine, David Geffen School of Medicine, University of California, Los Angeles, California, USA, Los Angeles, California; Division of Pediatric Infectious Diseases, Department of Pediatrics, David Geffen School of Medicine, University of California, Los Angeles, California, USA, Los Angeles, California; Univeristy of California, Los Angeles, Los Angeles, California, USA, Los Angeles, California; David Geffen UCLA School of Medicine, Los Angeles, CA

## Abstract

**Background:**

Respiratory infections in children are highly dynamic requiring frequent assessments of epidemiological trends to mitigate their impact on the population. We aimed to analyze the landscape of viral respiratory illnesses (VRIs) in a large metropolitan area in Southern California with a focus on the COVID-19 pandemic.

**Methods:**

We conducted a retrospective cohort study within the UCLA Health System, which evaluated children aged 0-5 years who received comprehensive respiratory viral panel (cRVP) testing during August to February of 2018 to 2023. Patient demographics, disease severity, and clinical course were specifically compared during the pandemic. Predictors of moderate-to-severe VRI were determined by multivariate logistic regression.

**Results:**

In total, 1,321 children with a median age of 2 years (44% female) underwent cRVP testing. A total of 753 positive subjects were identified during the study period. Rhinovirus (RV) was by far the most frequent virus identified in children across 5 years, even during the COVID-19 pandemic period, followed by respiratory syncytial virus (RSV). Along with RSV and human metapneumovirus, rhinovirus (RV) was associated with the highest disease severity across the years, particularly in the Hispanic/Latino ethnic group. Importantly, RV-driven respiratory disease occurred independently of co-infection with other viruses.

**Conclusion:**

RV was the most common viral pathogen in young children even during the height of the COVID-19 pandemic and was an independent driver of moderate-to-severe disease particularly in children with co-morbidities. Ethnic disparities were observed in disease severity, underscoring the need for targeted interventions, and heightened clinical vigilance in pediatric populations.

**Disclosures:**

**All Authors**: No reported disclosures

